# New insects feeding on dinosaur feathers in mid-Cretaceous amber

**DOI:** 10.1038/s41467-019-13516-4

**Published:** 2019-12-10

**Authors:** Taiping Gao, Xiangchu Yin, Chungkun Shih, Alexandr P. Rasnitsyn, Xing Xu, Sha Chen, Chen Wang, Dong Ren

**Affiliations:** 1 College of Life Sciences and Academy for Multidisciplinary Studies, Capital Normal University, 105 Xisanhuanbeilu Haidian District, 100048 Beijing, China; 2 Northwest Institute of Plateau Biology, Chinese Academy of Sciences, 23 Xinning Road, 810008 Xining, China; 3 Department of Paleobiology, National Museum of Natural History, Smithsonian Institution, Washington, DC 20013-7012 USA; 4 A. A. Borissiak Palaeontological Institute, Russian Academy of Sciences, Moscow, Russia 117647; 5 Natural History Museum, Cromwell Road, London, SW7 5BD UK; 6 Key Laboratory of Vertebrate Evolution and Human Origins, Institute of Vertebrate Paleontology and Paleoanthropology, Chinese Academy of Sciences, 100044 Beijing, China; 7 Center for Excellence in Life and Paleoenvironment, Chinese Academy of Science, Beijing, China; 8 School of Health Administration and Education, Capital Medical University, No.10 Xitoutiao, You An Men, 100069 Beijing, China

**Keywords:** Palaeoecology, Palaeontology, Taxonomy, Entomology

## Abstract

Due to a lack of Mesozoic fossil records, the origins and early evolution of feather-feeding behaviors by insects are obscure. Here, we report ten nymph specimens of a new lineage of insect, *Mesophthirus engeli* gen et. sp. nov. within Mesophthiridae fam. nov. from the mid-Cretaceous (ca. 100 Mya) Myanmar (Burmese) amber. This new insect clade shows a series of ectoparasitic morphological characters such as tiny wingless body, head with strong chewing mouthparts, robust and short antennae having long setae, legs with only one single tarsal claw associated with two additional long setae, etc. Most significantly, these insects are preserved with partially damaged dinosaur feathers, the damage of which was probably made by these insects’ integument-feeding behaviors. This finding demonstrates that feather-feeding behaviors of insects originated at least in mid-Cretaceous, accompanying the radiation of feathered dinosaurs including early birds.

## Abstract (web summary)

Numerous feathered dinosaurs and early birds have been discovered from the Jurassic and Cretaceous, but the early evolution of feather-feeding insects is not clear. Here, Gao et al. describe a new family of ectoparasitic insects from 10 specimens found associated with feathers in mid-Cretaceous amber.

## Introduction

Many extant insects have ectoparasitic lifestyles, and spend lots of time or entire life (obligate) on the skin, hairs, or feathers of warm-blooded vertebrates, sucking blood or feeding on skin debris, hairs, or feathers of their hosts^1^. Ectoparasitic insects cause discomforts or sickness of hosts, reduce production of livestock, and damage avian feathers, etc^2,3^. More seriously, ectoparasites transmit diseases as vectors^4^, having resulted in catastrophic illnesses and deaths in human history^3,5^. Two major groups of extant ectoparasites, Phthiraptera (true lice), and Siphonaptera (fleas), have attracted much attention from scientific community mainly due to their medical and agricultural significance. One lineage of the former group feed on feathers and soft skin of birds^6,7^. While blood-feeding insects have been described from the Jurassic and Cretaceous^8–10^, integument-feeding insects have never been reported from the Mesozoic to our knowledge. The earliest known fossil louse, *Megamenopon rasnitsyni*, is from the Eocene of Germany (44 Mya)^11,12^, and it is already fully modern in form and assigned within Amblycera. The evolution of feather- and other integument-feeding insects in the Mesozoic thus remains obscure^11,13^, even though many feathered dinosaurs including early birds have been described from the Jurassic and Cretaceous^14^.

Here, we report ten nymph specimens of an ectoparasitic insect clade, *Mesophthirus engeli* Gao, Shih, Rasnitsyn & Ren, gen et. sp. nov. assigned to Mesophthiridae Gao, Shih, Rasnitsyn & Ren, fam. nov. of order Incertae sedis. These nymph insects crawled and fed on two feathers preserved in two pieces of amber, AMBER No. 01 and AMBER No. 02, from the mid-Cretaceous of Myanmar. AMBER No. 01 includes nine specimens and AMBER No. 02 has only one specimen. The strata producing the Myanmar (Burmese) amber was radiometrically dated at 98.79 ± 0.62 Ma^15^. The date corresponds to the early Cenomanian, that is, the earliest Late Cretaceous. However, displaying clear traces of redeposition, the Myanmar amber is considered to be older than enclosing rocks^16^. Therefore, we refer the amber age informally as mid-Cretaceous.

## Results

### Systematic paleontology

Insecta Linnaeus, 1758

Order Incertae sedis

Family Mesophthiridae Gao, Shih, Rasnitsyn & Ren, fam. nov.

**Fig. 1:**
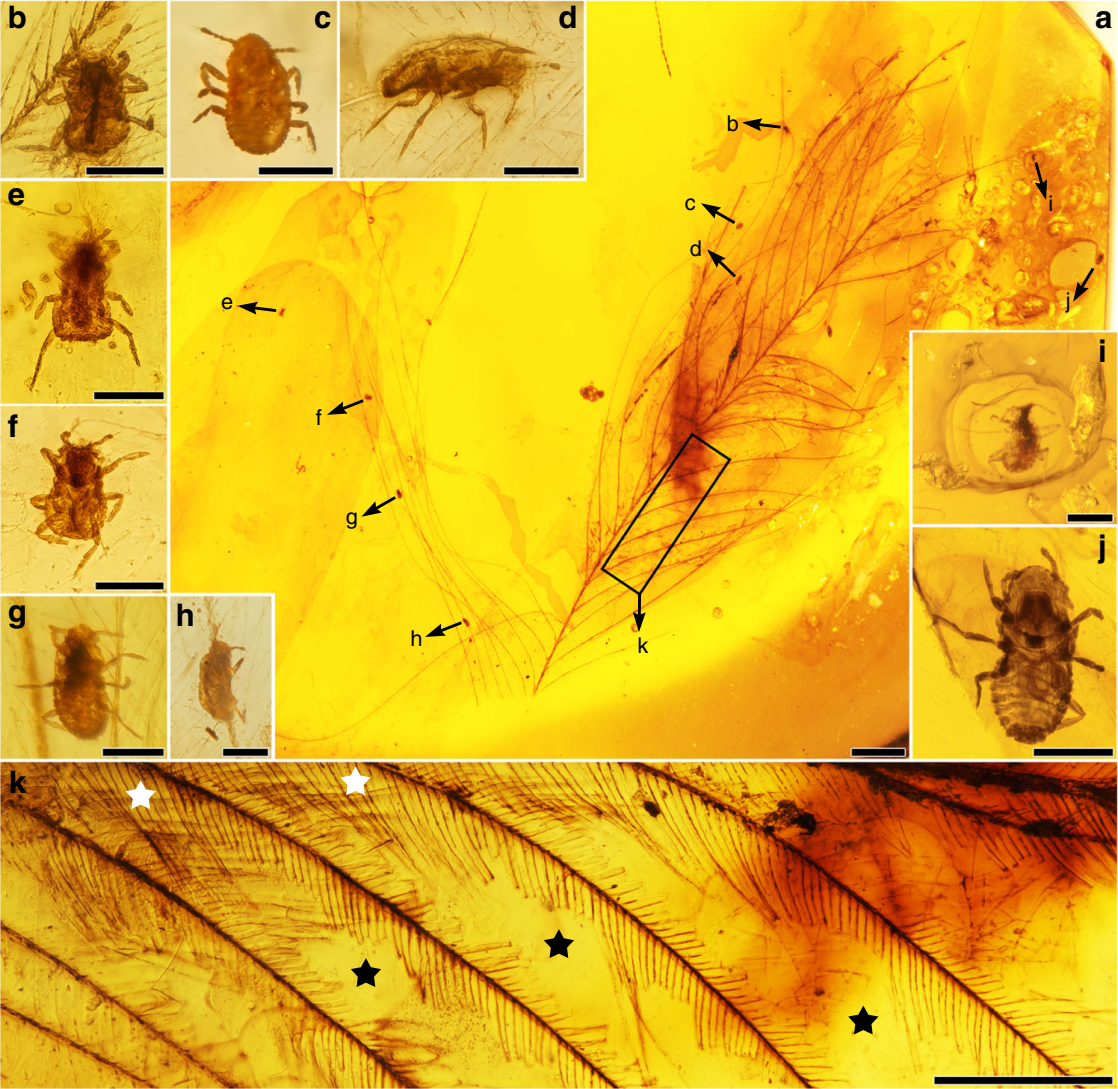
AMBER No. 01 with the specimens of *Mesophthirus engeli* Gao, Shih, Rasnitsyn & Ren, gen. et sp. nov. from the mid-Cretaceous of Myanmar. **a** Photo of the whole feather and the locations of the insects, corresponding to the Supplementary Fig. 1. **b**–**i** Paratypic specimens CNU-MA2016001 to CNU-MA2016008. **j** Holotypic specimen CNU-MA2016009. **k** Parts of the feather show complete areas at basal part and adjacent largely damaged area between barbs. Representative, star in white referring to relatively complete barbules, star in blank referring to large areas of damages. Scale bars, 1 mm (**a**), 100 μm (**b**–**j**), and 0.5 mm (**k**).

**Fig. 2:**
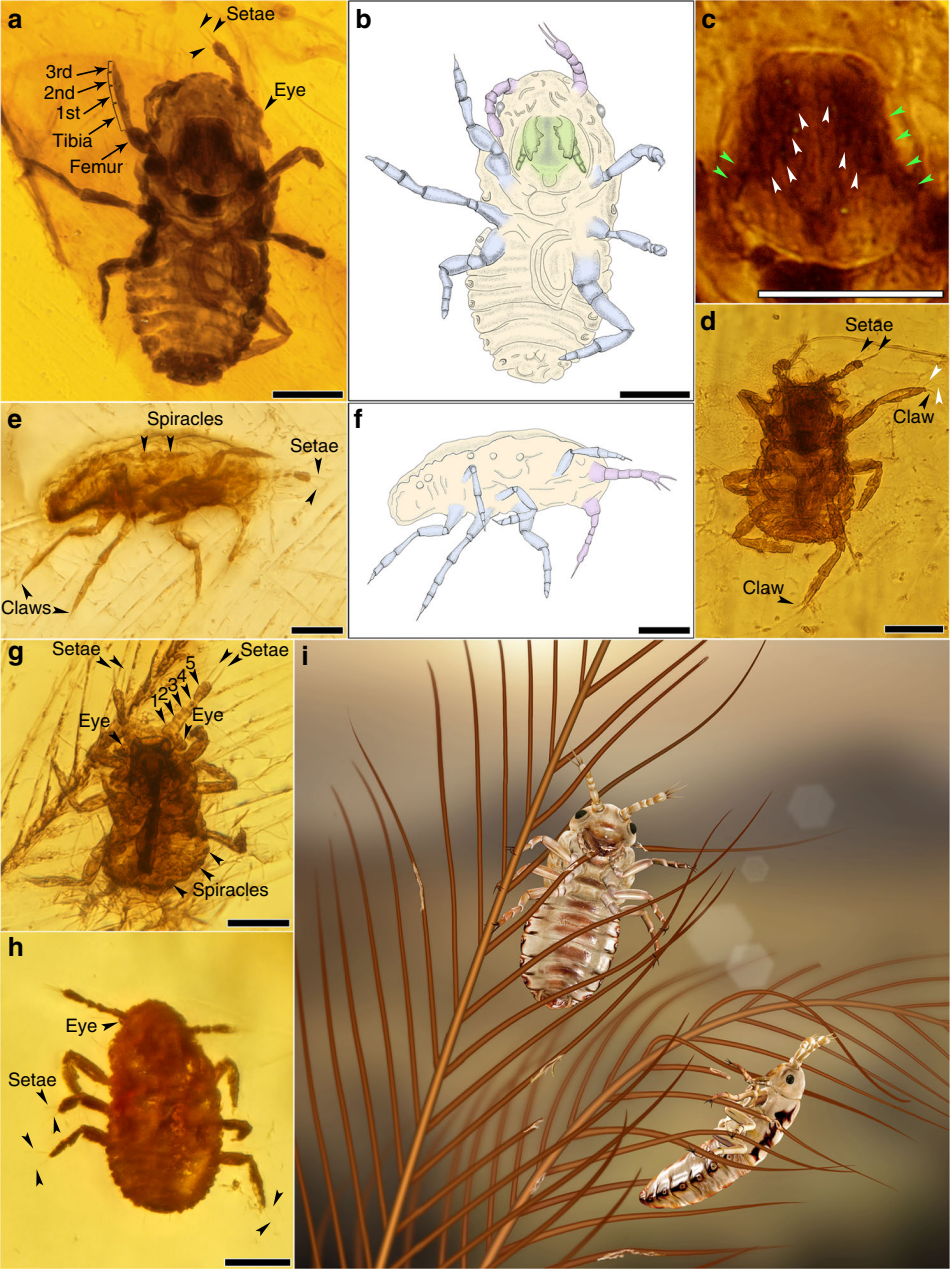
Holotype and paratypes of *Mesophthirus engeli* Gao, Shih, Rasnitsyn & Ren, gen. et sp. nov. within the AMBER No. 01 from mid-Cretaceous. **a**, **b** Holotype and line drawing of *M. engeli* sp. nov., CNU-MA2016009. **c** Enlargement of mouthpart of a shows details of mandible and maxillary palpi, arrows in white show teeth and arrows in green show the segments of palpi. **d** Paratype of *M. engeli* sp. nov., CNU-MA2016005, shows setae on the apex of antenna and single pretarsal claw. **e**, **f** Paratype and line drawing of *M. engeli* sp. nov., CNU-MA2016003, show spiracles on meso- and metanotum. **g** Paratype of *M. engeli* sp. nov., CNU-MA2016001, shows details of antennae, eyes, and spiracles on the side of abdominal segments, indicating the size scale of *M. engeli* sp. nov. with a feather barbule. **h** Paratype of *M. engeli* sp. nov., CNU-MA2016002, shows the two long stiff setae on the outsides of pretarsus. **i** Artist’s reconstruction of *M. engeli* sp. nov. of elder development stage feeding on the feather. It was reconstructed mainly based on the morphological characters of the holotype CNU-MA2016009, with supplemental consideration of CNU-MA2016001 and CNU-MA2016003. Colors of the insects are conjectural and referring to the general color of living feather-feeding lice. Scale bars, 50 μm.

**Fig. 3:**
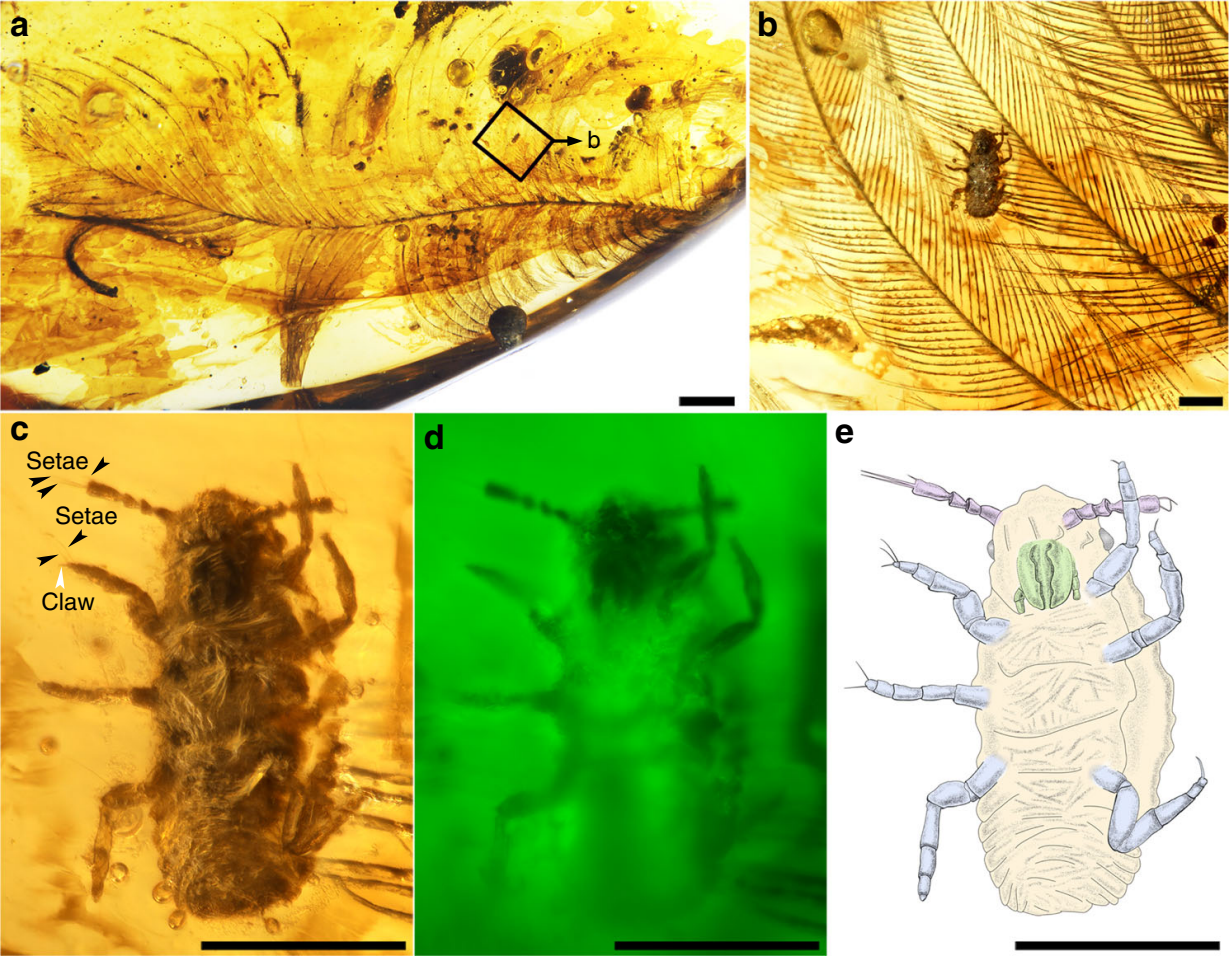
AMBER No. 02 with the paratype of *Mesophthirus engeli* Gao, Shih, Rasnitsyn & Ren, gen. et sp. nov. from the mid-Cretaceous of Myanmar. **a** Photo of the whole feather and the location of the insect. **b** Enlargement of the insect crawling on the feather. **c** Paratype of *M. engeli* sp. nov., CNU-MA2016010. **d** CNU-MA2016010 under green epifluorescence. **e** Line drawing of **c**. Scale bars, 1 mm (**a**), 0.1 mm (**b**–**e**).

**Type genus**. *Mesophthirus* Gao, Shih, Rasnitsyn & Ren, gen. nov. (Figs. 1–3)

**Diagnosis**. Based only on nymphs of elder developmental stages, while the adults unknown. Small insects, body wingless and dorsoventrally flattened, head fully hypognathous, slightly narrower than thorax; eyes reduced; huge mandible with at least four teeth; maxillary palp four-segmented; antenna with five antennomeres, last antennomere slightly enlarged, with two long setae and one short seta extending from apex, last antennomere longer than sub two antennomeres combined; thorax equal to abdomen in length, with pronotum distinctly longer than, and about as wide as mesonotum; tarsus with three tarsomeres, as long as tibia, basitarsus as thick as tibia, last (3rd) tarsomere very small, possessing a claw, and a pair of long and stiff setae.

**Genera included**. Only the type genus, *Mesophthirus* Gao, Shih, Rasnitsyn & Ren, gen. nov., described here.

**Remarks**. Comparing with Phthiraptera, Mesophthiridae shows several plesiomorphies: toothed chewing mouthpart, robust thorax with pronotum not reduced in length and width, leg with tarsus 3-segmented and spiracles present on meso- and metathorax as well as on the first two abdominal segments. However, several putative morphological synapomorphies are shared by these Cretaceous specimens: antenna with two apical long stiff setae and one short seta, leg with a single claw accompanied with two long clavate pretarsal setae. Based on these characters, it is difficult to place Mesophthiridae to any known order within Insecta, therefore we identify the taxon as an order incertae sedis here, pending more new material to be found in the future to further confirm its phylogenetic position. For more details concerning possible taxonomic position of the new family, see “Discussion” section.

Mesophthirus engeli Gao Shih, Rasnitsyn & Ren, sp. nov. (Figs. 1–3, Supplementary Figs. 1 and 2)

**Type species**. *Mesophthirus engeli* Gao, Shih, Rasnitsyn & Ren, sp. nov.

**Etymology**. The generic name is derived from the Greek prefix “meso-” and Greek “phtheir” (latinized as phthirus) meaning lice. Gender masculine. The specific name “engeli” is dedicated to Dr Michael S. Engel, for his outstanding contribution to entomological research.

**Species included**. Only the type species *Mesophthirus engeli* Gao, Shih, Rasnitsyn & Ren, sp. nov.

**Diagnosis**. As for family because of monotypy.

**Materials**. All specimens are nymphs of two different developmental stages (see “Discussion” section below). The holotype, CNU-MA2016009 (Fig. 1j and Fig. 2a–c), together with remaining six paratypes, CNU-MA2016001–CNU-MA2016003 (Fig. 1a–c), CNU-MA2016006—CNU-MA2016008 (Fig. 1g–i) and CNU-MA2016010 (Fig. 3b–e) are in an elder stage, and paratypes CNU-MA2016001 (Fig. 1a), CNU-MA2016004 (Fig. 1e) and CNU-MA2016005 (Fig. 1f) are in an earlier stage. The locations of CNU-MA2016001—CNU-MA2016009 with feather are displayed in Fig. 1, and more details about the morphology of this group are shown in Fig. 2. All these specimens are deposited in the Key Lab of Insect Evolution and Environmental Changes, Capital Normal University, Beijing, China.

**Locality and horizon**. The amber specimen was collected from Kachin Province (Hukawng Valley) of northern Myanmar, mid-Cretaceous; see “Methods” section for more information about provenance of the specimens.

### Description

Holotype, CNU-MA2016009 (Figs. 1a, j, and 2a–c). Small nymph, just 229 µm long excluding antenna (Table 1), but the largest one among these ten specimens. Head slightly thinner than the thorax in width, the length about 1/3 of the body length. Compound eyes reduced to single ommatidium, near globular shape, evidently protruding from both sides of head. Ocelli absent. Antenna short (46 μm) and robust, slightly shorter than the width or length of head, with five antennomeres. Antennal scape slightly enlarged, shorter than pedicel, the first flagellomere shortest and thinnest, and the last or 5th one longest. Two long stiff setae, longer than the last flagellomere, together with a short seta, fixed on the apex of the antenna (Fig. 2a, b). No annulations or other setulae visible. Chewing mouthpart area very clear, mandibles slightly extended vertically, enlarged and having four sharp teeth (Fig. 2b, c). The left and right mandibles apparently can cross with each other. Maxillary palpus with four segments and tapered from base to top (Fig. 2c). Thorax equal to abdomen in width, but shorter than the latter. Pronotum clear, about as wide as, and distinctly longer than either mesonotum or metanotum. Legs short, about 92, 115, and 134 μm, referring to fore leg, middle leg, and hind leg, respectively. Fore legs with coxae enlarged and nearly bulb shaped. Trochanter visible. Femora oval-shaped, much thicker and slightly longer than tibiae. Three tarsomeres present, basitarsus longest, the 3rd or last tarsomere very small (Fig. 2a, b). The femora of middle legs absolutely longer than those of fore legs or hind legs. Abdomen at least eight segmented, and the first segment narrow, connecting with metathorax and forming a clear constriction between thorax and abdomen. The 4th segment widest and gradually tapered to the terminal. Spiracles presented on both sides of each abdominal segment. Adults unknown.

**Table 1:** Dimensions of all ten specimens from mid-Cretaceous (µm).

No.	Body excluding antennae	Antenna excluding setae	Fore legs	Mid legs	Hind legs
CNU-AM2017001	141	56	>42	>81	>95
CNU-AM2017002	167	51	>76	–	>71
CNU-AM2017003	203	56	90	107	>106
CNU-AM2017004	143	51	>69	>57	>119
CNU-AM2017005	143	44	>88	>89	>119
CNU-AM2017006	198	50	>90	>88	>111
CNU-AM2017007	183	>37	>73	–	>86
CNU-AM2017008	>156	59	>100	–	>108
CNU-AM2017009	229	46	92	115	134
CNU-AM2017010	216	44	>70	100	>80

### Descriptions of the paratypes

Additional nine specimens of paratypes are shown in (Figs. 1–3, Supplementary Figs. 1 and 2), and more detailed descriptions are given in Supplementary Note 1. In CNU-MA2016001 (about 141 μm in body length excluding antenna, Figs. 1b and 2g), CNU-MA2016004 (about 143 μm, Fig. 1e and Supplementary Fig. 2a) and CNU-MA2016005 (about 143 μm, Figs. 1f and 2d), the last antennomeres have irregular crinkling on the surface. The apical parts of their heads between two antennae, are horizontal rather than convex and arch-shaped of that of the holotype or other paratypes. In addition, these three specimens possess much fatter and softer abdomens, and are faintly delimited about the boundaries of each segments. In CNU-MA2016002 (Figs. 1c and 2h), CNU-MA2016005 (Figs. 1f and 2d), and CNU-MA2016010 (Fig. 3), only one pretarsal claw is found on the apex of every tibia (Figs. 2d, h and 3c). Two very long stiff setae protrude outside from the base of the pretarsus, and the length of these setae approximately equal to whole tarsi (Fig. 3c). The boundaries among pronotum, mesonotum, and metanotum are distinct in CNU-MA2016010 (Fig. 3c–e). In CNU-MA2016003 (Figs. 1d, 2e, f), we can clearly see the dorsoventrally compressed body shape and spiracles are present on the meso- and metathorax, and consequently on the sides of abdominal segments (Fig. 2e). The tergum is strongly sclerotized, which could be observed in CNU-MA2016003 (Fig. 2e, f) and CNU-MA2016010 (Fig. 3c–e).

## Discussion

All these ten nymphs look very similar in morphology but have minor distinctions, and they may be easily divided to two different groups. The first group includes CNU-MA2016001 (Figs. 1b and 2g), CNU-MA2016004 (Fig. 1e and Supplementary Fig. 2a), and CNU-MA2016005 (Figs. 1f and 2d), and the remaining ones, including the holotype, can be arranged as the second group. The members of the first group have relatively smaller body size (under 145 μm, Table 1), much wider abdomens than their thoraxes, horizontal terminals of abdomens and apex of heads and compressed 5th antennomere. In contrast, the specimens within the second group possess bodies over 157 μm in length, heads with arched top and clavate and smooth 5th antennomere. In addition, the abdomens of these insects within the second group gradually tapered toward to terminal, and the boundaries between each abdominal segment are much clearer than those of the first group. We identify these two groups as two different but adjoining developmental stages of *Mesophthirus engeli*, and their abdomens and antennae might grow to extend with age, same as the body size. We estimate that the adults of *M. engeli* should be significantly larger than these nymphs, about 0.4–0.5 mm in body lengths.

*Mesophthirus* shares many features of ectoparasitic function, i.e., wingless and dorsoventrally compressed body, reduced eyes, short antennae, robust and short legs unsuitable for quick movement or jump, pretarsus very small with one single claw, etc., which suggest that *Mesophthirus engeli* Gao, Shih, Rasnitsyn & Ren, gen. et sp. nov. had an ectoparasitic lifestyle. As aforementioned, these ten specimens are preserved in close association with two feathers in two pieces of amber, respectively. The two feathers are probably contour feathers, but they differ in some features. The feather embedded in AMBER No. 01 (Fig. 1a and Supplementary Fig. 1a) is 13.6 mm long as preserved and is missing the proximal half. The feather has a proportionally slender rachis and two vanes of similar size. The barbs are positioned alternately along the rachis, and barbules are present along both barb ramus and rachis (rachidial barbules). These features are also seen in the feather of probably a nonpennaraptoran coelusaurian dinosaur preserved in a Myanmar amber^17^. There are nine insects of *M. engeli* found in AMBER No. 01 (Fig. 1a and Supplementary Fig. 1a). Five of them (Fig. 1c, e, f, i and j) are preserved near the feather and the other four insects are preserved with the feather (Supplementary Fig. 1). CNU-MA2016001 and CNU-MA2016003 are preserved on the vane of the feather, with feather debris around CNU-MA2016001 (Fig. 2e and g). CNU-MA2016006 (Fig. 1g and Supplementary Fig. 2b) is preserved with its legs hooking two adjoining feather barbs with the assistance of antenna (Supplementary Fig. 2b). In addition, feather debris are found around CNU-MA2016004 (Supplementary Fig. 2a) and CNU-MA2016008 (Supplementary Fig. 2d). CNU-MA2016007 is preserved with its legs tightly hugged a feather barb (Fig. 1h and Supplementary Fig. 2c). The feather of AMBER No. 02 (Fig. 3a) is nearly complete, missing the basal portion and the tip, with the preserved rachis length of 12.7 mm. It is clearly pennaceous as indicated by a robust rachis and closed vanes characterized by the pennaceous arrangement of the barbules, but it is not a flight feather based on the following features: the spacing of the neighboring barb rami is relatively wide and the distal portion of the vanes is open. This feather is probably derived from a pennaraptoran dinosaur^18^. CNU-MA2016010 is positioned in the proximal portion of the vane (Fig. 3a, b), more specifically, on the interlocking barbules between two rami.

The feather within AMBER No. 01 has multiple regions showing damages (Fig. 1a, k), though no damage has been observed in the other feather in AMBER No. 02 (Fig. 3a). Numerous neighboring barbules are missing their distal portion of variable length, resulting in many holes in both the leading and trailing vanes of the feather (Fig. 1k), which is in a stark contrast to the basal part close to rachis when the barbules are preserved intact, suggesting that this damage was caused by insect chewing instead of a damage due to use. Associated with the large toothed mandible of *Mesophthirus* (Figs. 2a–c and **3**c–e), these damaged areas might be interpreted as caused by integument-feeding behaviors by these insects. The association of the specimens of *Mesophthirus* with feathers within two different pieces of amber and the clear consumption–damage strongly suggest that *Mesophthirus* is ectoparasitic, living, and feeding on feathers (Fig. 2i). The damaged areas of the feather in the AMBER No. 01 are very similar to the consumed areas or holes of bird feathers caused by living lice^19,20^. These two feathers are significantly different from each other in general morphology, suggesting that they might belong to different dinosaur groups. If this is true, *Mesophthirus* was probably not host-specific as extant lice or fleas. However, these two feathers are possible to be derived from the same dinosaur species given that feathers are highly variable in morphology across the avian body. Nevertheless, the new findings indicate that the feather-feeding behavior of insects appeared at least as early as the mid-Cretaceous (Fig. 2i).

Since the new taxon of *Mesophthirus* is established based on only nymphs, it is difficult to place *Mesophthirus* within an accurate modern insect order, even though two developmental instars were described here. *Mesophthirus* shares several putative synapomorphies (or possibly, homoplasies) with Liposcelidae + Phthiraptera for their apterous and dorsoventral flattening body, reduced eyes, short antenna under ten antennomeres^21,22^. However, different from living lice (Phthiraptera)^6,7^, *Mesophthirus* specimens have a few putative plesiomorphies, such as four-segmented maxillary palpus, spiracles on meso- and metathorax and on the first two abdominal segments, and relatively long and wide pronotum. *Mesophthirus* is also noteworthy for the two stiff and long setae on pretarsi and three clavate setae on the apex of antenna, but no other setae are found on all these ten specimens. Such specialized setae distinctly helped *Mesophthirus* to hold on to the feathers of their hosts avoiding being shaken off (Figs. 1a, g, 2g and Supplementary Fig. 2b), which are attributed to adaptations to ectoparasitic habit. Therefore, it is prudent to only provisionally assign these specimens from the mid-Cretaceous to *Mesophthirus engeli* Gao, Shih, Rasnitsyn & Ren, gen et. sp. nov. within Mesophthiridae Gao, Shih, Rasnitsyn & Ren, fam. nov. of order Incertae sedis, while more material, especially the adults to be found in the future, will further help to define the exact phylogenetic position.

In summary, the new findings provide the earliest known evidence about the origin of ectoparasitic insects feeding on feathers, which strongly support that the integument-feeding behaviors of insects appeared during or before the mid-Cretaceous along with the radiations of feathered dinosaurs including birds.

## Methods

### Localities and repositories

This fossil study included two pieces of amber with ten specimens of insects. Both pieces of amber were collected from the Hukawng Valley of Kachin State, in northern Myanmar, a village named Noije Bum (N26°150′, E96°340′) about 18 km southwest of the town of Tanai^23,24^. The amber is dated as earliest Upper Cretaceous, about 98.79 ± 0.62 Ma^15,24^. The study of Burmese amber has a long history going back over 100 years, and over 1000 species of insects had been named by the end of 2018^25^, including beetles^26^, ants^27^, termites^28^, lacewings^29^, etc. The amber specimens were acquired by Mr Fangyuan Xia in 2015, who donated these two pieces of amber for studying in 2016. All type specimens are permanently deposited at the Key Lab of Insect Evolution and Environmental Changes, College of Life Sciences, Capital Normal University, Beijing, China (CNUB; Dong Ren, Curator) under the collection number CNU-MA2016001—CNU-MA2016010.

### Amber preparation

The amber piece of AMBER No. 01 is nearly ovoid, about 2.6-cm long and 1.9-cm wide, but very thin (about 5 mm in maximum). The nine insects it contains are preserved in various position and distributed in different places and layers within this piece of amber, and dissection or polish may easily destroy this amber and insects. Only the left side of this amber was trimmed by razor blade, then polished with emery papers and diatomite mud. For observing the specimen of CNU-MA20160010, AMBER No. 02 was trimmed to a subtriangular chip, and the edges are about 0.9 mm in maximum.

### Specimen imaging

We have tried to use Confocal Laser Scanning Microscopy and MicroXCT even SRμCT to observe more morphological characters, but with no success. It might be caused by the fact that these insects are too small and nearly semitransparent. Photographs were taken by using a Nikon SMZ 25 microscope with a Nikon DS-Ri 2 digital camera system, and the enlarged images of details of the specimens were taken using a Nikon ECLIPSE Ni microscope with a Nikon DS-Ri 2 digital camera system. Photographs with green background were taken using green epifluorescence as the light source attached to Leica DM5500B with an ANDOR Zyla digital camera system (Fig. 3d). Line drawings were prepared by using Adobe Illustrator CC and Adobe Photoshop CC graphics software.

### Nomenclatural acts

This published work and the nomenclatural acts it contains have been registered in ZooBank, the proposed online registration system for the International Code of Zoological Nomenclature (ICZN). The ZooBank LSIDs (Life Science Identifiers) can be resolved and the associated information viewed through and standard web browser by appending the LSID to the prefix “http://zoobank.org/”. The LSIDs for this publication are urn:lsid:zoobank.org:pub: F962A768-8075-4319-BBFC-BE438209533D (for publication); urn:lsid:zoobank.org:act:3040E401-99AD-4257-8784-58A46448884F (for Mesophthiridae fam. nov.); urn:lsid:zoobank.org:act:AE0355E2-5A56-48DB-8A1A-74F4FAC2ED8D (for *Mesophthirus* gen. nov.); urn:lsid:zoobank.org:act:0439DE1E-84BE-48D6-AEAB-147C2B13631D (*Mesophthirus engeli* sp. nov.).

### Reporting summary

Further information on research design is available in the Nature Research Reporting Summary linked to this article.

## Supplementary information

Supplementary Information

Reporting Summary

## Data Availability

All data needed to evaluate the conclusions are present in the paper and/or the Supplementary Information files. All data related to this paper are available from the corresponding authors upon reasonable request.
