# Supplementary material for: New insects feeding on dinosaur feathers in mid-Cretaceous amber
Source: Nat Commun. 2019 Dec 10;10:5424. doi: 10.1038/s41467-019-13516-4 (PMC6904634; doi:10.1038/s41467-019-13516-4)
Supplement: Supplementary file 1 — Supplementary Information [file 41467_2019_13516_MOESM1_ESM.pdf]

Supplementary Information

New insects feeding on dinosaur feathers in mid-Cretaceous amber

Gao et al.

This PDF file includes:

- Supplementary Fig. 1. Details of the feather within AMBER No. 01 showing locations of the specimens CNU-MA2016001 to CNU-MA2016009.
- Supplementary Fig. 2. Enlarged photos of paratypes of *Mesophthirus engeli* Gao, Shih, Rasnitsyn & Ren, gen. et sp. nov. embedded in AMBER No. 01.
- Supplementary Note 1: Descriptions of the paratypes of *Mesophthirus engeli* Gao, Shih, Rasnitsyn & Ren, gen. et sp. nov.

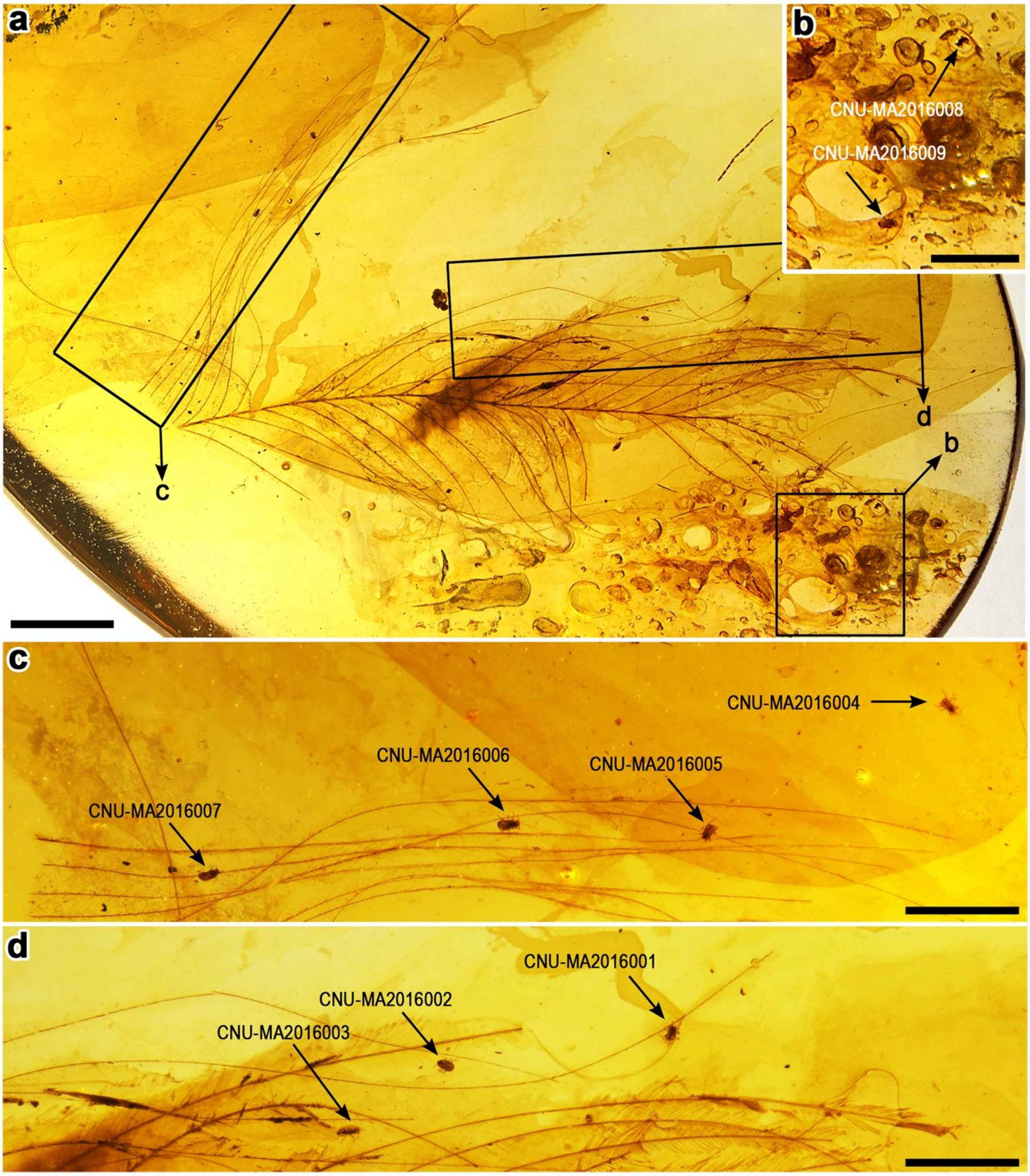

Supplementary Figure 1 | Details of the feather showing locations of the specimens CNU-MA2016001 to CNU-MA2016009. (**a**) Photo of the AMBER No. 01 with feather. (**b**) Locations of specimens of CNU-MA2016008 and CNU-MA2016009. (**c**) Locations of specimens of CNU-MA2016004 to CNU-MA2016007. (**d**) Locations of specimens of CNU-MA2016001 to CNU-2016003. Scale bars, 2.0 mm (**a)** and 1.0 mm, (**b**–**d**).

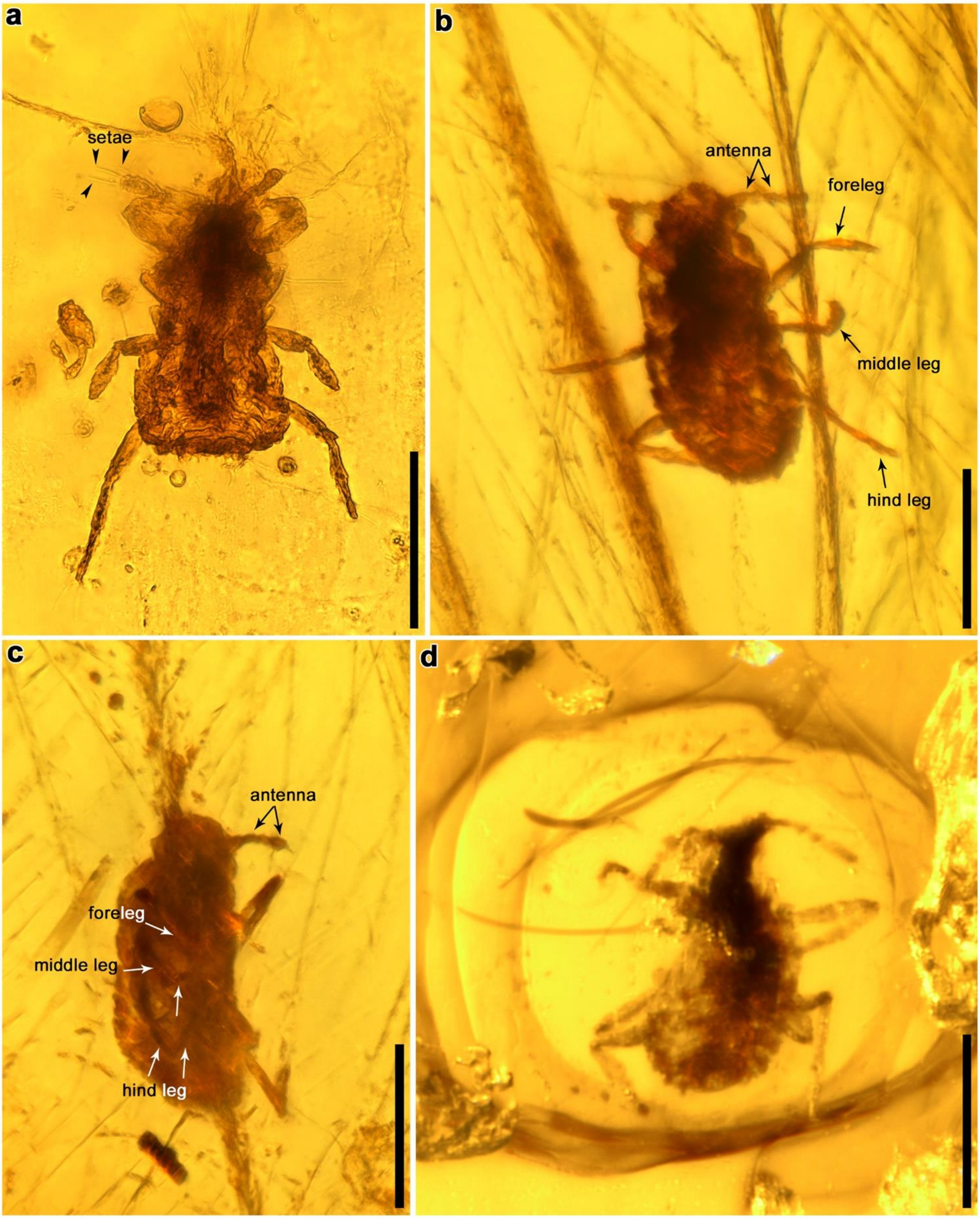

Supplementary Figure 2 | Enlarged photos of paratypes of *Mesophthirus engeli* Gao, Shih, Rasnitsyn & Ren, gen. et sp. nov. embedded in AMBER No. 01. (**a**) Specimen CNU-MA2016004 referring to Fig. 1e. (**b**) Specimen CNU-MA2016006 referring to Fig. 1g. (**c**). Specimen CNU-MA2016007 referring to Fig. 1h. (**d**). Specimen CNU-MA2016008 referring to Fig. 1i. Scale bars, 100 µm (**a**–**d**).

Supplementary Note 1:Descriptions of the paratypes of *Mesophthirus engeli* Gao, Shih, Rasnitsyn & Ren, gen. et sp. nov.

Paratype, CNU-MA2016001, the earlier developmental stage of *Mesophthirus engeli* Gao, Shih, Rasnitsyn & Ren, gen. et sp. nov. (Figs 1b and 2g, Supplementary Fig. 1d).

Very tiny insect, crawling on the surface of feather, closer to the barb of feather. Details of the characters are indistinct since the specimen overlapped with the barbules and other feather fragments. Body only 141 μm in length excluding antenna. Head slightly thinner than thorax, and the division between head and thorax unclear. Eyes ovoid, nearly protruding out of the sides of the head symmetrically. Antennae robust and longer than the width of head (56 μm), thinner than the adjacent feather barbs but thicker than the barbules. Scape broader than pedicel, and the latter thicker than the 3rd antennomere. The 4th antennomere is the thinnest one, about 1/3 as long as the 5th or last antennomere, which is nearly claviform with clear constrictions. Only two long and sharp setae visible and fixed on the apex of the antenna, and can easily hold the feather barbule observed from the left antenna (Fig. 2g). Thorax longer than abdomen, but thinner than the latter. Prothorax, meso- and metathorax fused and the divisions difficult to be identified. The bases of legs distantly spaced. Profemur slightly shorter than mesofemur, and the latter shorter than metafemur, but nearly similar in thicknesses. The combination of mesotibia + mesotarsus nearly equal to that of metatibia + metatarsus in length. Abdomen soft and bilaterally extended, the boundaries between each segment ambiguously, and spiracles present on both sides of the abdomen, especially clear on the right side. The body has a rough surface without other setae. Irregular feather fragments scattered around this specimen.

Paratype, CNU-MA2016002, the elder developmental stage of *Mesophthirus engeli* Gao, Shih, Rasnitsyn & Ren, gen. et sp. nov. (Figs 1c and 2h, Supplementary Fig. 1d)

Body about 167 μm in length, preserved away from the feather (Supplementary Fig. 1d). Head thinner than thorax, and the top of the head outward, forming an arched-shape. Antenna relatively thin, only 4 antennomeres visible in dorsal view. Pedicel cylindrical, and the 1st and 2nd flagellomeres nearly caliciform. The apical antennomere longest. Two stiff setae, slightly shorter than the last antennomere, extended from the apex. Thorax connected with head and abdomen tightly, but the boundaries unclear. The profemur nearly equal to mesofemur or metafemur in length. In hind legs, femur longer than tibia, and the latter equal to tarsus in length. Three tarsomeres present, protarsus very small but having two very long stiff setae protruding out of outside surface. These two setae apart from each other for a long distance. Abdomen with 6 segments visible, tapering from the basal segments to terminal.

Paratype, CNU-MA2016003, the elder developmental stage of *Mesophthirus engeli* Gao, Shih, Rasnitsyn & Ren, gen. et sp. nov. (Figs 1d, 2e and 2f, Supplementary Fig. 1d)

This specimen is the only one observed in the right lateral view. Body about 203 μm high, dorsoventrally compressed. The back of the whole body clearly sclerotized, including tergum and part of the head. Head fully hypognathous, having dimly edge toward to thorax. Antenna with the 4th antennomere thinnest but equal to front antennomeres in length. The 5th or last flagellomere possessing three apical stiff setae, and two of them much longer, equal to the last flagellomere in length. Scape thicker then pedicel, and as thick as the last antennomere. The divisions of pronotum, meso- and metanotum obscured in the lateral view. Profemur thicker than protibia, and equal to the tarsus in length. The protarsus slightly longer than tibia, three tarsomeres tapered. Mesocoxae elongated, and the apical part clearly thinner than the basal part. Instead, the mesofemora and mesotibiae calycinal. Metacoxae and metatibiae slightly longer than those of middle legs. Two plate-like spiracles present on the side of mesothorax and metathorax. Abdomen short, 8 segments visible, and the terminal bending inward. Spiracles distributed on the side of each abdominal segment.

Paratype, CNU-MA2016004, the earlier developmental stage of *Mesophthirus engeli* Gao, Shih, Rasnitsyn & Ren, gen. et sp. nov. (Fig. 1e, Supplementary Figs. 1c and 2a)

The specimen was preserved with a similar gesture as CNU-MA2016001, and two fore legs crossed in the front of head. Many feather fragments were found around its head. Body about 143 μm in length. Head with the antenna compact. Five antennomeres visible; the 4th one shortest, and the last one longest with many clear constrictions. Three stiff setae fixed on the top of the last flagellomere, two long and one short. Thorax clearly thinner than abdomen. Profemur thicker than mesofemur, than metafemur. Mesotibia cup-shaped, thicker but much shorter than that of hind legs. A pair of long and stiff setae present on the outside of the pretarsi of every middle and hind leg. Abdomen soft and lateral extending, but the boundaries of segments unclear.

Paratype, CNU-MA2016005, the earlier developmental stage of *Mesophthirus engeli* Gao, Shih, Rasnitsyn & Ren, gen. et sp. nov. (Figs. 1f and 2d, Supplementary Fig. 1c)

Body about 143 μm in length, as high as the specimen CNU-MA2016004. Head with the eyes nearly spherical, clearly protruding out of the sides of head. The top part of the head between two antennae horizontal. Antenna with 5 antennomeres visible and compact, scape very thick, the 4th antennomere smallest and shortest, cup-shaped. The

5th or last flagellomere having many constrictions, and deformed. Thorax wider than head but clearly thinner than abdomen. In the ventral view, the divisions of prothorax, meso- and metathorax unclear, and fused together, but can be identified from the lateral sides. Bases of the left and right legs distantly spaced. Forelegs almost equal to middle legs, but slightly shorter than the hind legs. Protibia as long as combination of three tarsomeres together. The basitarsus close to cylindrical, shorter than the 2nd tarsomere, and the pretarsus very small, with only a sharp claw, which is slightly longer than the basitarsus. Two long and stiff setae protruding outside of basal part of the pretarsus, and both of them twice as long as the pretarsal claw. Abdomen bilaterally extending, the boundaries of the abdominal segments unclear. The terminal of the abdomen bending inward.

Paratype, CNU-MA2016006, the elder developmental stage of *Mesophthirus engeli* Gao, Shih, Rasnitsyn & Ren, gen. et sp. nov. (Fig. 1g, Supplementary Figs. 1c and 2b)

A specimen crawling on feather and clearly holding two adjoining barbs via legs with assistance of antennae. Body about 198 μm high, with the head distinctly thinner than thorax or abdomen. The compound eyes right under antennae. Antenna as long as the width of head, and the part of head between two antennae prominent as an arch. Antenna with the 3rd and 4th antennomeres cup-shaped, equal in length. The thorax fused with the abdomen so that the division unclear. Abdominal segments cannot be distinguished, but tapered toward to the terminal.

Paratype, CNU-MA2016007, the elder developmental stage of *Mesophthirus engeli* Gao, Rasnitsyn & Ren, gen. et sp. nov. (Fig. 1h, Supplementary Figs. 1c and 2c)

The specimen distinctly wrapping a feather barb, left foreleg, left middle leg and left hind legs bending and holding the barb respectively; right foreleg and middle leg holding the same barb too. Body about 183 μm in length. The top part of head between antennae arched. The details of the thorax, legs and abdomen unclear. The terminal of abdomen slightly bending inward, and tightly attaching the barb.

Paratype, CNU-MA2016008, the elder developmental stage of *Mesophthirus engeli* Gao, Shih, Rasnitsyn & Ren, gen. et sp. nov. (Fig. 1i, Supplementary Figs. 1b and 2d)

A specimen far away from the feather, but close to the specimen CNU-MA2016009, and several feather fragments distributing around. Body at least 156 μm in length. Head with the antenna longer than the width of head. Head thinner than thorax, and the latter thinner than abdomen. Profemur slightly shorter than the combined lengths of tibia and tarsus. Metafemur longer than metatibia. Metatibia cup-shaped, equal to the tarsus.

Paratype, CNU-MA2016010, the elder developmental stage of *Mesophthirus engeli* Gao, Shih, Rasnitsyn & Ren, gen. et sp. nov. (Fig. 3)

This specimen was found in the other piece of amber, AMBER No. 02, and provided more evidence and further confirmed the feather-feeding behaviors of *Mesophthirus engeli* gen. et sp. nov. CNU-MA2016010 is the second largest specimen among these new findings, the body about 216 μm in length, just smaller than the holotype CNU-MA2016009. Head fully hypognathous, slightly thinner than thorax, with the eyes protruding from both sides of head. Antenna shorter than the width or length of head, with 4 antennomeres visible, scape and pedicel relatively thick, the 2nd–4th antennomeres pedunculate, having relatively long stipe; the last antennomere club-shaped with smooth surface, and the terminal two setae very stiff, as long as the last antennomere (Fig. 3c). The top part of the head between the antennae arched, with the surface rough. Chewing mandible with the teeth structures unclear, maxillary palpus at least two segments visible. Thorax very large, equal to abdomen in length, clearly divided as prothorax, mesothorax and metathorax, no fusion, and the surface rough and scraggly, pronotum longest, reaching eyes (Fig. 3a and 3b). Legs robust. Profemur nearly equal to protibia or protarsus, but thicker than the latter. Tarsus including three tarsomeres, the basitarsus and the 2nd tarsomere thick and long, 3rd one very small, possessing one claw and two long clavate setae. The stiff and long setae as long as the tarsus and extending from the outside of protarsus, close to the basal part. The metafemur longer that mesofemur or profemur. The space between left legs and right legs very broad (Fig. 3d). Abdominal segments totally compacted, especially in the ventral view, difficult to identify the boundaries, but over 7 segments, the 1st segment widest, and gradually tapering off toward the terminal, the abdomen slightly bending to the ventral side.
