# Supplementary material for: New insects feeding on dinosaur feathers in mid-Cretaceous amber
Source: Nat Commun. 2019 Dec 10;10:5424. doi: 10.1038/s41467-019-13516-4 (PMC6904634; doi:10.1038/s41467-019-13516-4)
Supplement: Supplementary file 2 — Reporting Summary [file 41467_2019_13516_MOESM2_ESM.pdf]

natureresearch

Corresponding author(s): Dong Ren

Reporting Summary

Nature Research wishes to improve the reproducibility of the work that we publish. This form provides structure for consistency and transparency in reporting. For further information on Nature Research policies, see Authors & Referees and the Editorial Policy Checklist.

Statistical parameters

When statistical analyses are reported, confirm that the following items are present in the relevant location (e.g. figure legend, table legend, main text, or Methods section).

n/a	Confirmed	
☐	☒	The exact sample size (n) for each experimental group/condition, given as a discrete number and unit of measurement
☐	☒	An indication of whether measurements were taken from distinct samples or whether the same sample was measured repeatedly
☒	☐	The statistical test(s) used AND whether they are one- or two-sided<br>*Only common tests should be described solely by name; describe more complex techniques in the Methods section.*
☒	☐	A description of all covariates tested
☒	☐	A description of any assumptions or corrections, such as tests of normality and adjustment for multiple comparisons
☒	☐	A full description of the statistics including central tendency (e.g. means) or other basic estimates (e.g. regression coefficient) AND variation (e.g. standard deviation) or associated estimates of uncertainty (e.g. confidence intervals)
☒	☐	For null hypothesis testing, the test statistic (e.g. F, t, r) with confidence intervals, effect sizes, degrees of freedom and P value noted<br>*Give P values as exact values whenever suitable.*
☒	☐	For Bayesian analysis, information on the choice of priors and Markov chain Monte Carlo settings
☒	☐	For hierarchical and complex designs, identification of the appropriate level for tests and full reporting of outcomes
☒	☐	Estimates of effect sizes (e.g. Cohen's d, Pearson's r), indicating how they were calculated
☒	☐	Clearly defined error bars<br>*State explicitly what error bars represent (e.g. SD, SE, CI)*

Our web collection on statistics for biologists may be useful.

Software and code

Policy information about availability of computer code

Data collection	No other special software used.
Data analysis	No other special software used.

For manuscripts utilizing custom algorithms or software that are central to the research but not yet described in published literature, software must be made available to editors/reviewers upon request. We strongly encourage code deposition in a community repository (e.g. GitHub). See the Nature Research guidelines for submitting code & software for further information.

Data

Policy information about availability of data

All manuscripts must include a data availability statement. This statement should provide the following information, where applicable:

- Accession codes, unique identifiers, or web links for publicly available datasets
- A list of figures that have associated raw data
- A description of any restrictions on data availability

The authors declare that the data supporting the findings of this study are available within the paper and its supplementary information files. All relevant data are available from the corresponding author upon request. The nomenclatural acts of new taxon have been registered in ZooBank, the proposed online registration system for the International Code of Zoological Nomenclature (ICZN). The LSIDs (Life Science Identifiers) for this publication are: urn:lsid:zoobank.org:pub:

F962A768-8075-4319-BBFC-BE438209533D (for publication); urn:lsid:zoobank.org:act:3040E401-99AD-4257-8784-58A46448884F (for Mesophthiridae fam. nov.); urn:lsid:zoobank.org:act:AE0355E2-5A56-48DB-8A1A-74F4FAC2ED8D (for Mesophthirus gen. nov.); urn:lsid:zoobank.org:act:0439DE1E-84BE-48D6-AEAB-147C2B13631D (Mesophthirus engeli sp. nov.)

Field-specific reporting

Please select the best fit for your research. If you are not sure, read the appropriate sections before making your selection.

- [x] Life sciences
- [ ] Behavioural & social sciences
- [ ] Ecological, evolutionary & environmental sciences

For a reference copy of the document with all sections, see nature.com/authors/policies/ReportingSummary-flat.pdf

Life sciences study design

All studies must disclose on these points even when the disclosure is negative.

Sample size	This study included 2 amber with 10 fossil specimens.
Data exclusions	No data excluded from the analysis.
Replication	We make sure this study can be repeated under the same conditions.
Randomization	All the specimens are described here and the holotype selection is not random. These specimens preserved in different position with varied gestures lead to a better understanding of this group of insects.
Blinding	All the observed morphological characters of these specimens are figured out and described comparably.

Reporting for specific materials, systems and methods

Materials & experimental systems

n/a	Involved in the study	
[x]	[ ]	Unique biological materials
[x]	[ ]	Antibodies
[x]	[ ]	Eukaryotic cell lines
[ ]	[x]	Palaeontology
[x]	[ ]	Animals and other organisms
[x]	[ ]	Human research participants

Methods

n/a	Involved in the study	
[x]	[ ]	ChIP-seq
[x]	[ ]	Flow cytometry
[x]	[ ]	MRI-based neuroimaging

Palaeontology

Specimen provenance	This fossil study included two pieces of amber with 10 specimens of insects. Both pieces of amber were collected from the Hukawng Valley of Kachin State, in northern Myanmar, a village named Noije Bum (N26º150´, E96º340´) about 18 km southwest of the town of Tanai. The amber is dated as earliest Upper Cretaceous, about 98.79 ± 0.62Ma. The study of Burmese amber has a long history going back over 100 years, and over 1000 species of insects had been named by the end of 2018, including beetles, ants, termites, lacewings, etc. The amber specimens were acquired by Mr. Fangyuan Xia in 2015, who donated these two pieces of amber for studying in 2016. All type specimens are permanently deposited at the Key Lab of Insect Evolution and Environmental Changes, College of Life Sciences, Capital Normal University, Beijing, China (CNUB; Dong Ren, Curator) under the collection number CNU-MA2016001 – CNU-MA2016010.
Specimen deposition	All type specimens are housed at the Key Lab of Insect Evolution and Environmental Changes, College of Life Sciences, Capital Normal University, Beijing, China (CNUB; Dong Ren, Curator).
Dating methods	The amber was dated as earliest Upper Cretaceous, about 98.79 ± 0.62Ma based on the reference paper of "Shi, G. et al. Age constraint on Burmese amber based on U–Pb dating of zircons. Cretac. Res. 37, 155–163 (2012)."

- [x] Tick this box to confirm that the raw and calibrated dates are available in the paper or in Supplementary Information.
